# Emerging Applications of Nanotechnology in Healthcare Systems: Grand Challenges and Perspectives

**DOI:** 10.3390/ph14080707

**Published:** 2021-07-21

**Authors:** Sumaira Anjum, Sara Ishaque, Hijab Fatima, Wajiha Farooq, Christophe Hano, Bilal Haider Abbasi, Iram Anjum

**Affiliations:** 1 Department of Biotechnology, Kinnaird College for Women, Lahore 54000, Pakistan; saraishaque117@gmail.com (S.I.); hfatima13@gmail.com (H.F.); wajihafarooq93@gmail.com (W.F.); iram.anjum@kinnaird.edu.pk (I.A.); 2 Laboratoire de Biologie des Ligneux et des Grandes Cultures (LBLGC), INRAe USC1328, Université d’Orléans, 28000 Chartres, France; hano@univ-orleans.fr; 3 Department of Biotechnology, Quaid-i-Azam University, Islamabad 54000, Pakistan; bhabbasi@qau.edu.pk

**Keywords:** nanotechnology, nanosystems, healthcare, cancer, gene therapy, genetic disorders, drug delivery

## Abstract

Healthcare, as a basic human right, has often become the focus of the development of innovative technologies. Technological progress has significantly contributed to the provision of high-quality, on-time, acceptable, and affordable healthcare. Advancements in nanoscience have led to the emergence of a new generation of nanostructures. Each of them has a unique set of properties that account for their astonishing applications. Since its inception, nanotechnology has continuously affected healthcare and has exerted a tremendous influence on its transformation, contributing to better outcomes. In the last two decades, the world has seen nanotechnology taking steps towards its omnipresence and the process has been accelerated by extensive research in various healthcare sectors. The inclusion of nanotechnology and its allied nanocarriers/nanosystems in medicine is known as nanomedicine, a field that has brought about numerous benefits in disease prevention, diagnosis, and treatment. Various nanosystems have been found to be better candidates for theranostic purposes, in contrast to conventional ones. This review paper will shed light on medically significant nanosystems, as well as their applications and limitations in areas such as gene therapy, targeted drug delivery, and in the treatment of cancer and various genetic diseases. Although nanotechnology holds immense potential, it is yet to be exploited. More efforts need to be directed to overcome these limitations and make full use of its potential in order to revolutionize the healthcare sector in near future.

## 1. Introduction

Nanobiotechnology, a recently coined term, emerged from the blending of molecular biology and nanotechnology. It is a branch of science which revolves around structures or functional materials at the nanoscale, which are produced by employing both physical and chemical methods [1]. In the last thirty years, the discipline of nanotechnology has been a crucial area of research, due to the unique chemical, electrical, optical, biological, and magnetic properties of nanomaterials [2]. Nanotechnology has managed to attract a lot of attention, because it is an established fact that when nanotechnology joins hands with biotechnology, they give birth to a platform which holds immense potential and importance with respect to diversity in applications [3]. Some of these applications include medical imaging, diagnostic kits, diagnostic assays, biological sensors, dentistry, sterilization of medical device surfaces, sunscreens, cosmetics, sports equipment, textiles, environmental cleanup, and gene inactivation [1,4,5]. The development of nanotechnology has provided mankind with some incredible tools that allow the delineation of processes to a degree which was considered to be next to impossible a few years ago [6].

Various types of nanoparticles (NPs), such as metal, metal oxide, semiconductor, organic, and inorganic NPs, have been synthesized in order to exploit their properties. They can be formed via different procedures such as conventional chemical production and green synthesis processes [7]. Associated with toxicity, cost and efficiency, chemically produced NPs pose many problems. Thus, because of their ease of production, low cost and toxicity, bio-inspired NPs hold an edge over traditionally produced NPs [8]. The high cost of raw materials, drug wastage, chemical and physical incompatibilities, clinical drug interactions, and the occurrence of side effects associated with the dose, are the vital limitations of conventional approaches [9].

Generally, NPs range in size from 1 to 100 nm but some exceptions also exist [10]. For example, in medicine, NPs range in size from 5 to 250 nm [11]. There are also some nanosystems that may exceed several micrometers in size, e.g., liposomes. The definition and classification of NPs are continuously evolving as this field is progressing day and night. Adapting the technical and translational information on nanomaterials and nanotechnology from the US National Nanotechnology Initiative and European Commission, the authors feel that it is imperative to mention that the upper size limit of NPs cannot be restricted to 100 nm [12]. In fact, some commercial nanomedicine products are greater than 100 nm, e.g., abraxane (130 nm) and Myocet (180 nm). Therefore, we can limit or specify the range of nanomaterials only on the basis of their sizes [11].

Exceptionally small sizes enable NPs and nanodevices to exhibit novel properties and functions. It should be kept in mind that the small size of NPs gives them another advantage, perhaps their main advantage, which is that they have a very high surface area-to-volume ratio. This may sound trivial but this property actually makes them more reliable and reproducible [13]. In addition, they show enhanced catalytic activity, chemical stability, and thermal conductivity and non-linear optical performance [3]. Various NPs can be developed into nanosystems via modifications in their shape, surface properties, and size to efficiently utilize them in the imaging, diagnosis, and treatment of serious diseases. Controlled released therapy can be provided by means of these functionalized nanomaterials which send drugs to particular sites or tissues [14]. In order to optimize and promote tissue and cell interaction, some factors, such as charge, size, the pattern of nanoscale medical molecules, and shape, need to be modulated and investigated [15].

Nanotechnology products have become increasingly useful in healthcare and have led to the advent of novel nanosystems for the diagnosis, imaging, and treatment of various diseases, such as cancer, as well as cardiovascular, ocular, and central nervous system-related diseases [16,17,18]. Nanomaterials integrate well into biomedical devices because most biological systems are also nanosized [5]. In the field of drug delivery, nanosystems offer the precise delivery of drugs to the target tissues or organs with a controlled release and enhanced retention time as compared to conventional techniques. Nano-liposomes are one of the best examples of the nanosystems currently developed for targeted drug delivery to treat various types of cancers and cardiovascular diseases [9,14]. Drug delivery to target tissue, good biocompatibility, and the control of drug flow in the bloodstream are the most significant reasons for the usage of nano-liposomes [9].

Advents in nanomedicines and nanodevices has inspired numerous researchers to look for alternative therapies, as the currently employed methods are limited in terms of earlier detection and treatments. The astonishing properties and applications of various nanomaterials and nanosystems have made them pervasive in the development of technologies to be implemented in the near future. The purpose of this review is to provide readers with information about the most recent applications of nanotechnology in various healthcare sectors in one place. Furthermore, we also critically discuss the limitations, challenges, and future prospects of nanotechnology in allied healthcare systems.

## 2. Nanosystems Used in Various Healthcare Sectors

Nanotechnology revolves around some common nanostructures, no matter what field or area of application is concerned. Some of the important ones are nanoparticles, carbon nanotubes, dendrimers, nanoprobes, quantum dots, nano-diamonds, and nanowires (Table 1). Nanoparticles possess unique characteristics and their strikingly small size makes them able to cross microscopic pores and membranes easily. Nanoparticles are broadly classified into five categories, including metal, lipid, ceramic, polymeric, and semi-conductor NPs. Metal NPs are made out of metal precursors. These in particular have unique optoelectrical properties [19]. Ceramic NPs are inorganic and nonmetal NPs are found in amorphous, polycrystalline hollow and dense forms [20]. They are efficient catalysts and help in the photodegradation of dyes and imaging technologies [21]. Semiconductor NPs have properties of both metal and non-metal NPs; hence, they also find applications in numerous fields such as photo-optics and electronic devices [22,23]. Polymeric NPs are organic NPs, which are either matrix particles—that are generally solid which can adhere to molecules to be transported—or are encapsulated within the particle [24]. Lipid NPs contain moieties that are lipid in nature. These are usually spherical in shape and diameters range from 10 to 100 nm. They have a solid lipid core and lipophilic molecules can be transported easily.

Carbon nanotubes (CNT) are nanosized, seamless tubes made out of graphite sheets. They have open terminal parts that are closed by fullerene caps. They have the highest mechanical strength out of all natural materials. They are efficient absorbers of magnetic radiation, along with providing the efficient conduction of heat and having catalyzing properties. Their properties are dependent on their purity, length and diameter, special surface area, and amorphous carbon. Carbon nanotubes are included in the fullerene nanotube family and have a rather cylindrical configuration. CNTs also include buckyballs, which are spherical and cylindrical in shape [25]. CNTs are widely employed in modern healthcare systems because they have the potential to overcome hindrances that were previously impossible to address. They can cross partially permeable cell membranes very easily, using a mechanism that is still unclear. They can carry small organic drugs, proteins, peptides, nucleic acids, antibiotics, etc., to precise locations. These small molecules can be either covalently attached, adsorbed, or encapsulated in these CNTs [26]. They can carry protein less than 80 KDa that can be bound either covalently or non-covalently. These are taken up by cells via endocytosis. CNTs also have applications in X-ray imaging [27]. A CNT solution was placed in a laser infrared beam, which was able to heat CNTs up to 158 °F in 2 min. Cells containing CNTs are not destroyed by laser beams since they can absorb near-infrared waves. These lasers can effectively kill cancer cells [28].

Dendrimers are naturally biodegradable nanopolymers. They are macromolecular nanostructures having a 3D globular shape due to the presence of many branched layers. Their small size (1–10 nm), globular structure, and the fact that they can penetrate through cell membranes due to their lipophilic nature make them ideal systems for use in healthcare for gene and drug delivery purposes [28,29,30]. A dendrimer structure consists of three major components—the core made of an atom or a multifunctional molecule, repetitive branching units covalently bound to the core, and many functional groups present at the terminal of the branching units [31,32]. Dendrimers interact with drugs through physical and chemical interactions. The physical interactions (encapsulation of the drug) are due to the presence of empty internal cavities, which bind the drug molecules through hydrophobic interactions [33,34,35]. The chemical interactions occur either through electrostatic interactions (due to the presence of ionizable functional groups in dendrimers) or through covalent bonding [36]. For covalent binding, the dendrimer surface is first mixed with active moieties such as poly-ethylene glycol (PEG) or p-amino benzoic acid, etc. After this, the drugs can successfully conjugate with the dendrimers through covalent bonding [32,37].

Nano-diamonds (NDs) are nanostructures consisting of a single diamond crystal with carbon in the sp^3^ configuration. Their particle size is approx. 4–5 nm. NDs are very hard and chemically inert and they have high thermal conductivity and bio-compatibility [38]. They have a tunable surface and a large surface area to which drugs and genes can easily conjugate. The fluorescence produced by NDs makes them useful as imaging probes for diagnostic purposes [39,40]. All these properties of NDs are actually due to the combined characteristics of diamonds and NPs [40]. The structure of NDs consists of two major components—(1) the inner diamond core, with carbon atoms in the sp^3^ configuration; and (2) the outer graphitic shell (carbon atoms in the sp^2^ configuration), with functional groups on the terminal of dangling bonds [41]. Techniques used for the synthesis of NDs include the detonation of explosives, high temperature, high pressure, and the chemical vapor deposition method [22].

Quantum dots are synthetic nanostructures ranging in size between 1.5–10 nm. their semi-conductor nature allows them to transport electrons. When UV light passes through them, the electrons in the QDs are excited, and when these excited electrons move back to their ground state, they emit light. QDs emit light of different colors depending upon their size [42]. QDs made from heavy metals such as cadmium are very toxic and carcinogenic; therefore, they cannot be widely used in the health sector. However, graphene and carbon QDs are safe and stable and have wide scope in the health sector [43].

Nanofilms consist of polymeric sheets with a large surface area and a thickness of relatively few nanometers (10–100 nm) [44]. Multiple oppositely charged layers are assembled together to form multilayered yet ultra-thin biofilms. Layers are deposited one by one for deposition. Various methods are used for the deposition of individual layers, including fluidic assembly, electromagnetic deposition, spin coating, and emersion [45].

**Figure 1:**
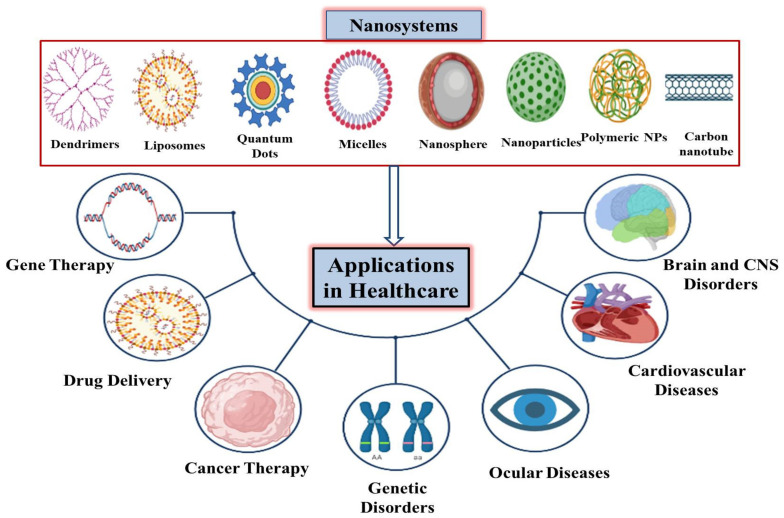
Schematic presentation of applications of various nanosystems in allied healthcare sectors.

Liposomes are spherical vesicles made up of one or more lipid bilayers with an aqueous compartment in between them [42,43]. They are found in a variety of sizes, starting from as small as a few nanometers, and can be as large as several micrometers [44]. They are capable of entrapping various substances, including hydrophilic and lipophilic agents. Therefore, they are also considered to be the most efficient drug delivery system. Another reason for this is because their composition is very similar to the cellular membranes found in the body, which helps with drug delivery in vitro. Their large size also enables them to deliver a high quantity of drugs [45]. The major domains of healthcare in which nanotechnology-mediated nanosystems are playing their positive role are summarized in Figure 1.

**Table 1:** Applications of various nanostructures in healthcare sectors.

Nanostructure	Applications in Health Sector	References
Nanoparticles	Used as antimicrobials and antifungals; used as sensors, as catalysts, and for imaging in diagnostics	[5,21]
Carbon Nanotubes	Used for delivering fibrinogen and bovine protein to cells; serve as vectors for gene delivery; and in the treatment of broken bones, osteoporosis, and breast cancer	[26,27,46,47,48,49]
Dendrimers	Used for diagnostic applications, for gene delivery, as anti-bacterial agents, as anticancer drugs, to improve vaccine formulations by acting as carriers of antigens, and in treating ocular diseases.	[32]
Nano-Diamonds	Used for the treatment of bone disease by targeted drug delivery (bone regeneration); used in imaging and therapy, in the early detection of cancer, and in the treatment of brain and breast cancers	[40,50,51]
Quantum Dots	Useful in diagnostics, real time in vivo bio-imaging, in controlling various diseases, intracellular tracking and therapeutic drug delivery, and to deliver siRNA for RNA interference	[52,53]
Nanofilms	Act as useful biological, chemical and nanomechanical sensors in electrochemical devices, used for controlled drug release, used as nanopatches after open surgery to close incisions	[43,44,45,54,55,56,57,58]
Liposomes	Used for drug delivery, capable of containing hydrophobic and hydrophilic drugs, protect drugs from chemical and enzymatic degradation, have the ability to encapsulate anti-tumoral drugs, for example, anthracyclines such as epirubicin, daunorubicin, and Dox, etc.	[59,60,61,62,63]

## 3. Applications of Nanotechnology in Healthcare Sectors

### 3.1. Role of Nanotechnology in Gene Therapy

Gene therapy is a procedure to replace a defective gene in the DNA (which is responsible for causing a disease) with a normal gene. The gene is usually inserted into the stem cells using a vector [64]. Stem cells have long life and a self-renewal ability; therefore, they are the most suitable targets for gene therapy [65]. The vector used should be highly specific and efficient in releasing the gene or genes of variable sizes. It should not be recognized as an antigen by the host immune system. The vector must have the ability to express the inserted gene throughout the life of that organism [66,67]. When the gene is correctly inserted into the cells, it inhibits and corrects the functions of the mutated gene and induces the normal functioning of cells [68,69].

Viral vectors have been used for years in gene therapy and are still being used. They can take over the host metabolic machinery for the synthesis of proteins that are coded by their DNA. Furthermore, their insertion in the host genome is very stable, and the transduced cells cause the long-term expression of the transgene. These are the properties that make them suitable for gene therapy [67,68]. Some common and efficient viral vectors include lentivirus, retroviruses, adenoviruses, etc. [67,69,70,71,72,73,74,75,76]. However, there are many risks associated with the use of viral vectors. These include the generation of an immune response, inflammation, and the occurrence of off-target changes in the host body. If the virus triggers the immune response, it not only makes the therapy less efficient but when the same virus enters the body the second time (with the desired gene inserted into its DNA), a secondary immune response occurs, which would rapidly kill the virus, making it impossible to use the same virus for gene therapy [77,78,79,80,81]. Inflammation caused by viral vectors can sometimes be very dangerous, as reported in a recent study in which a leukemic patient died when given a high dosage of adenovirus for gene therapy [82]. Virus virulent genes are deleted prior to therapy, which also compromises the integration and infection ability of viral vectors. Insertional mutagenesis can be life-threatening too, because sometimes these viruses (mostly retroviruses) insert DNA into the tumor-suppressing gene or the oncogenes, activating them to cause tumors in the host body. The selection of appropriate viruses for different body cells is another difficulty in the field of gene therapy. Moreover, viruses can also go through genetic changes with the passage of time, which can lead to other complications in the body [83]. These are some major concerns relating to viral gene therapies, and therefore these methods are not encouraged, and the world is now moving towards the use of nanostructures for gene therapy.

**Figure 2:**
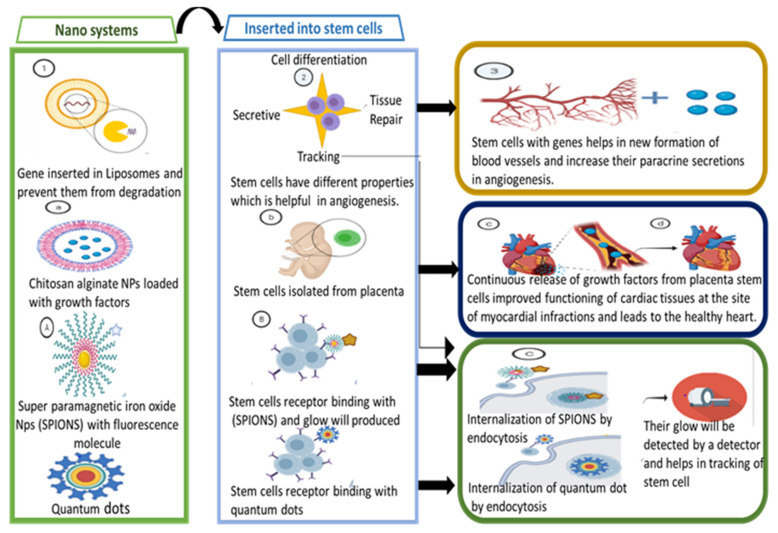
Graphical representation of various nanosystems used in gene therapy.

Gene therapy using non-viral nanostructures is safe, as compared to therapy using viral vectors. They are also much less oncogenic and rarely trigger immune responses. Their preparation is much easier than that of viral vectors. There is no risk of virus recombination and no limit on the size of the gene to be loaded. NPs are one of the many nanostructures that are used for non-viral gene delivery. The presence of a positive charge, small size, and high surface-to-volume ratio enables them to penetrate deep into the membranes, thus making them ideal vectors for gene delivery [84,85,86]. The major nanosystems used in gene therapy are shown in Figure 2.

One of the ways in which gene therapy treats many diseases is through gene silencing. Various diseases, such as autoimmune disorders, cancers, and viral infections, can be treated by silencing the expression of genes [87]. RNA interference using small interfering RNA (siRNA) has been used for gene silencing. SiRNA is a 21–25 nucleotide long double-stranded RNA molecule. It forms a complex with RNA-induced silencing complex (RISC) in the cytoplasm and targets the directed complementary mRNA molecule, thus silencing its expression [88,89].

This technique can be very useful if the problem of their stable delivery into the cytosol (they become unstable in physiological fluids) and limited intracellular uptake are resolved [90]. This problem can be resolved by using some vector system. Viral vectors are very risky to use, as mentioned earlier. However, non-viral NPs have been used to overcome these limitations [91]. For example, one of the over-expressed proteins in cancer cells is the RhoA protein. Anti-RhoA siRNA was encapsulated in chitosan-coated polyisohexylcyanoacrylate (PIHCA) NPs. When these NPs were administered to mice infected with breast cancer, they showed 90% tumor inhibition with no toxic effects [92].

In rheumatoid arthritis, tumor necrosis factor-α (TNF-α) plays a role in the release of cytokines and thus causes chronic inflammation. A nanocomplex, thiolated glycol chitosan (TGC) polymer loaded with poly-siRNA, was targeted to TNF-α, which proved to be very efficient in curing rheumatoid arthritis. The inhibition in bone erosion and a reduction in inflammation was also observed in mice in that study [93]. These are just a few examples; there are several other studies in which nanostructure-based complexes have been effectively used to deliver siRNA, thus treating various diseases.

In another way, genetic materials (RNA, DNA, siRNA) can be encapsulated or conjugated with NPs for efficient gene delivery [84,94,95,96]. The most efficient way to attach genes with NPs is through the formation of DNA-NP complexes. These complexes are formed by means of the electrostatic bonding between them. For this, the surface charge on the NPs is made positive, which then binds strongly with negatively charged nucleic acids. Liposomal and polymeric and many other nanostructures use this mechanism of gene transfer [85,97,98,99,100]. The encapsulation of genetic material in NPs protects them from enzymatic digestion when they are targeted into the cells. It also protects them from phagocytosis by monocytes [94]. Due to the advantageous aspects of nano-based gene therapy, research is in process on large scale to develop new strategies for its implementation in the healthcare sector.

### 3.2. The Role of Nanotechnology in Targeted Drug Delivery

Nanovectors have great potential in target-specific drug delivery for the treatment of various diseases. Targeted drug delivery is important, especially if the solvents of hydrophobic drugs are toxic. If these solvents are released somewhere else other than the target cell, they may enter the blood stream or other body fluids and contaminate them. Nanostructures allow the continuous controlled release of drugs in desired amounts. Specific and localized drug delivery also reduces drug doses. The small size of NPs allows them to penetrate deep into the tumor cells, and thus they can be useful in improving cancer treatments [94].

The NPs used for drug delivery must contain some important components, including a particle core, an outer biocompatible protective layer and a linking molecule for increased bioactivity (it attaches the core of NPs to bioactive molecules because of the reactive compounds present at both of its ends). Nanovectors are modified before drug delivery and this modification includes coating with ligands such as peptides, folic acid, and antibodies. Ligands are attached to NPs so that they can bind specifically to targeted sites to enhance the specificity even more [16,95,101,102,103,104,105,106]. It is essential to attach more than one ligand because if only one ligand is attached, there is a possibility that it may bind to receptors present in places other than on the targeted site. In addition, tumor cells are usually overexpress, i.e., they have more than one type of surface receptor [17].

Since nanovectors possess unique properties and various modifications can be performed during drug loading, scientists are now moving towards the implement of nanotechnology-based nanosystems for efficient targeted drug delivery with the aim of curing various serious diseases. Some examples of targeted drug delivery using nanovectors are discussed in the following sections.

### 3.3. Treating Cardiovascular Diseases through Nanosystems

Cardiovascular diseases cause millions of deaths around the world [18]. Various treatments have improved the survival rate of patients with heart diseases but none of them has achieved complete cardiac regeneration, especially for patients after cardiac infarction [107]. Stem cell therapy can be used for therapeutic angiogenesis [108]. Introducing anti-apoptotic and pro-angiogenic genes into the genetically engineered stem cells can prolong their rate of survival and increase their paracrine secretion [109,110]. Viral vectors cannot be used to deliver genes to stem cells as they cannot carry large gene volumes and have immunogenic effects. Bio-compatible NPs are efficient in transferring genes to stem cells. Various nanostructures can be used for delivering genes to stem cells. Liposomes are one of the best contenders for gene delivery as they can prevent the non-specific binding of genes and protect them from degradation [111,112]. Polymers show improved specificity for targets and higher efficiency [113]. In one study, chitosan alginate NPs were used to deliver growth factors to placental cells. The continuous release of growth factors improved the functioning of cardiac tissues at the site of myocardial infarction [114]. NPs also have the potential for tracking and monitoring stem cells. Superparamagnetic iron oxide nanosystems (SPIONs) are made to enter the cells by attaching to cell surfaces. These cells are then internalized by endocytosis [115]. Quantum dots can also be used for monitoring the living cells for a long time [116,117].

Hypertension is a disease that gives rise to many problems, including myocardial infarction, heart failure, stroke, increased blood pressure, and damage to many body organs, including the eyes, kidney, brain, etc. [118]. Many antihypertensive drugs have been used to treat this, but various problems are associated with the use of these drugs, including their short half-life, low bioavailability, poor solubility in water, unwanted side effects, and many more. Targeted drug delivery using nanostems has been effectively performed in order to solve these problems [119]. Nanocarriers that have been used so far for treating hypertension include lipid carrier NPs, solid lipid NPs, polymeric NPs, liposomes, and nanoemulsions [120]. These are just a few examples, but nanotechnology has very promising applications in treating many other cardiovascular diseases through non-viral stem cell-based therapies. Further studies on the effects of nanovectors in the cardiovascular system of a living model need to be performed before they can be safely used in humans.

### 3.4. Nanotechnology in the Treatment of Ocular Diseases

The efficient delivery of drugs in the eye is an enormous challenge because of the presence of complex barriers and elimination mechanisms in the eye. The various barriers present include the tear film, the ocular surface epithelium, and the internal blood–aqueous and blood–retinal barriers. NPs are, however, able to overcome these barriers because of their small size and highly variable surface properties. They can efficiently transport the drug to the targeted site with no toxic effects. Most of the NPs are biodegradable, which means they do not require surgical removal after they have delivered the drug [121,122].

Anterior eye diseases, such as cataracts, conjunctives, keratitis, dry eye, corneal injury, etc., are usually treated using eye drops but the corneal barrier causes drugs to have poor bioavailability. However, nanosystems can increase the bioavailability by prolonging the retention time of the drug on the surface of the eye and improving the penetration of the drug [123]. On the other hand, posterior eye diseases in the choroid and retina include retinoblastoma, glaucoma, choroidal neovascularization, macular degeneration, and posterior uveitis. Eye drops are not usually effective in treating these diseases, so interocular injections are performed, which leads to many unwanted side effects [124]. However, nanosystems have improved the delivery of drugs to the posterior portion of eye and the various nanosystems used for this purpose include nanovesicles, nanoimplants, NPs, and hydrogels [123].

### 3.5. Nanotechnology in the Treatment of Brain Diseases

Brain diseases can be treated efficiently if we can overcome the issue of the blood–brain barrier (BBB). The BBB is a boundary between circulating blood and the neural tissues of the brain. The presence of the BBB is the major hurdle in the treatment of brain diseases because it does not allow the drugs to enter the central nervous system (CNS) and maintains homeostasis in the brain. Any disturbance to the BBB causes neuro-inflammatory and neurodegenerative diseases such as Parkinson’s disease, Alzheimer’s disease, etc., but even a damaged BBB does not allow drugs to enter the brain [125,126]. However, various types of NPs can cross the BBB and so can efficiently deliver drugs to damaged areas of the brain. NPs use organic and inorganic materials as a core to penetrate the BBB. Inorganic materials include silica, molybdenum, cerium, iron, and gold, whereas organic materials that can be used include PLA, PLGA, and trehalose. The distinct features by which NPs are able to treat neurodegenerative diseases are their small size, high drug loading ability, and efficient imaging performance (particularly for inorganic NPs). Some NPs themselves show some therapeutic efficacy, i.e., showing antioxidant properties, inhibiting Aβ aggregation, and reducing ROS levels [125].

**Figure 3:**
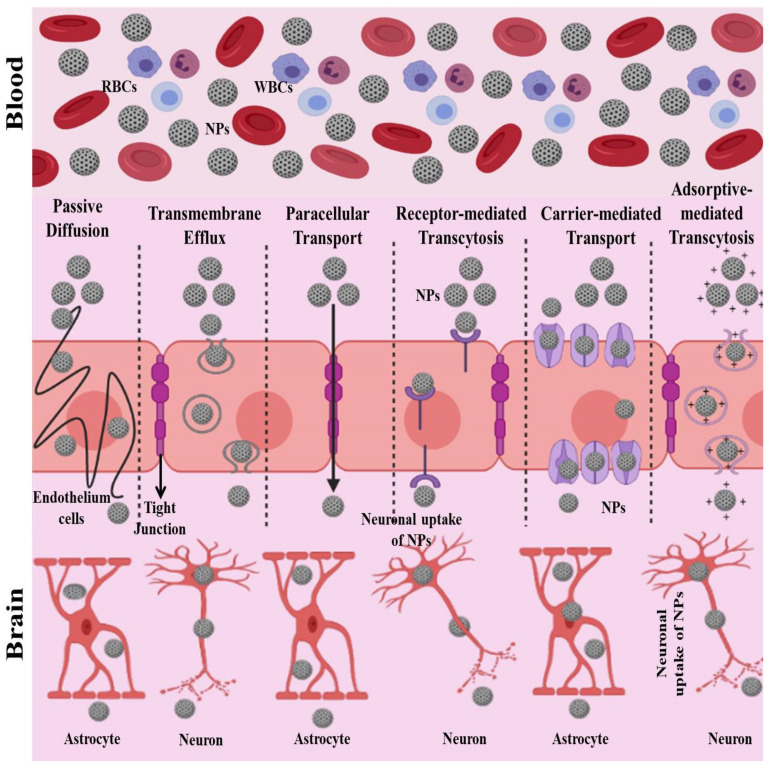
Schematic representation of the various proposed pathways by which NPs can cross the blood–brain barrier.

NPs, when conjugated with ligands, show the best performance by interacting with BBB receptors at low density. NPs can adopt multiple pathways in order to cross the BBB [127]. The proposed pathways which NPs can use to cross the BBB are shown in Figure 3. The main pathways include

- The paracellular pathway and passive transmembrane diffusion;
- Transport proteins: carrier-mediated transport and efflux proteins;
- Receptor-mediated transcytosis;
- Adsorptive-mediated transcytosis.

Through any of these pathways, NPs can cross the BBB and can be taken in by the neurons or active astrocytes of the brain [127,128]. In receptor-, adsorptive-, and carrier-mediated penetration of NPs across the BBB, various ligands are involved, such as:

- Ligands that can adsorb proteins from the bloodstream [129];
- Ligands that can directly interact with BBB transporters or receptors [130,131,132];
- Ligands that can increase the hydrophobicity and charge of NPs [133];
- Ligands that can improve the circulation time of NPs in the blood [133].

The morphology and charge of NPs are also important in this case. Zwitterion and neutral NPs have a greater circulation time compared to positively and negatively charged NPs [134,135]. Overcoming the blood–brain barrier has enabled NPs to be used for the treatment of many diseases such as stroke, Alzheimer’s, and Parkinson’s disease, and many more, which are discussed below.

### 3.6. Role of Nanotechnology in Cancer Diagnosis and Treatment

Nanomedicine involves the implementation of nanotechnology in the treatment, screening, and diagnosis of various diseases, including cancer, and has the potential to revolutionize public and individual health [134]. In the formulation of various drugs for cancer treatment and in the discovery of cancer biomarkers, nanotechnology plays a vital role [136,137]. Through prediction, personalized therapy, diagnosis, medicine, and the prevention of cancer, it also contributes comprehensive techniques and worthy approaches against cancer [138].

#### 3.6.1. The Utilization of Different Methods Involving Nanotechnology in Cancer Diagnosis

Obstacles in the early detection of different kinds of cancer are expected to be solved with the use of nanotubes, nanocantilevers, NP probes, and nanowire arrays [139]. Without the utilization of radioactive labeling or extrinsic fluorescent dyes, micro-cantilevers were used to determine single nucleotide polymorphisms in a 10-mer DNA target oligo-nucleotide [140]. Information about biomarkers related to the tumor microenvironment and the distribution, presence, and relative abundance of cancer signatures has been provided by probes with molecularly targeted recognition agents [139]. On the basis of fluorescence resonance energy transfer, nanoprobe systems have shown the potential to detect DNA mutations in tumor cells in clinical samples, as well as to detect the loss of DNA [141]. At the early stage of cancer, the expression of typical biological molecules has been detected using carbon nanotubes [142].

#### 3.6.2. Different Methods Used for Cancer Treatment

To ease the intake of vehicles into target cells, a number of cancer-targeting ligands, such as growth factors or folate, cytokines and antibodies, have been used [143]. Through enhanced permeability and retention effects, caplostatin (TNP-470) was found to aggregate selectively in the tumor vessels, as well as halting the hyper-permeability of cancerous blood vessels [144,145]. By means of the EPR effect, NP-conjugated chemo-therapeutic substances—for example, angiogenic minute molecule inhibitor and doxorubicin—can enter into cancer cells, causing a growth inhibition and particular vascular shutdown [146,147,148]. By enhancing imaging and targeting cancer cells, oligonucleotides perform an important function. Furthermore, coupling them to metallic NPs, e.g., quantum dots and magnetic, ruby-eye doped, and gold nanoparticles, increases their related vasculature [149,150,151,152]. For the direct observation of circulating tumor cells in blood, a new SERS NP system was also introduced recently [153].

#### 3.6.3. Targeting the Cancerous Micro-Environment 

The arginine-aspartic acid-glycine motif has been found to display strong selectivity and affinity for the cell surface in many proteins; therefore, it is an appropriate ligand for therapeutic NPs targeting cancer [154]. In an inactive form as a pro-drug or nanoformulation, a drug that has a short half-life or that is extremely cytotoxic in circulation may now be controlled. Through tumor-specific molecules, these drugs are targeted at the tumor micro-environment. The tumor micro-environment eases its transformation to an active state upon arriving at its destination. By attacking both stroma and tumor cells, this tumor-activated nanoformulation therapy performs its function [155].

Consisting of a lower dose of chemotherapeutic medicines, metronomic therapy includes an administered schedule which lacks long periods of rest [156,157]. The novelty of this concept is in targeting the tumor micro-environment, specifically endothelial cells that are highly sensitive to the continual administration of low dosages of medicine as compared to cancerous cells. Thus, tumor angiogenesis is inhibited, causing the inhibition of tumor growth [158]. Poly-base NPs, upon aggregation in the low-P^H^ micro-environment provided by the tumor tissue, become captured in the fenestrated cancerous vasculature and aid in the increased transfer of drugs to cancerous sites [159].

#### 3.6.4. Targeting Drug-Resistant Tumors

Metastatic colon tumor cells that overexpress integrin α5β1 have been found to be targeted by PEGylated liposomes altered with a fibronectin-mimetic peptide [160]. NPs have been made to increase endocytosis when targeting multidrug-resistant tumors, or to bypass or inhibit efflux pumps on the membrane, considering various methods of drug resistance in cancer cells [161]. As an effective inhibitor of P-gp, TPGS 1000 (d-alpha-tocopheryl polyethylene glycol 1000 succinate) has become one of the dominant surfactants that increases the cytotoxicity of the G-185 cells of colchicines, doxorubicin, paclitaxel, and vinblastine, which can be compared to that of parental cells [162]. Another promising and important example of a modifying substance for P-gb is the pluronic block copolymer (P85). In P85 treatment, membrane fluidization gives rise to an interference with metabolic mechanisms and the inhibition of the P-gb ATPase drug efflux system [163]. The NP system is made up of a lipid shell and a polylactic-co-glycolic acid (PLGA) doxorubicin-conjugated polymer core composed of cholesterol, PEG distearoylphos-phatidylethanolamine, and phosphatidylcholine. To cause vascular disruption in cancer cells, these NPs are filled with a natural phenolic compound called combretastatin [164]. 

To be overexpressed on the surface of drug-resistant cancer cells, NPs with folate acid ligands that can attach to folate receptors were shown to obtain particular accumulations in cancerous cells. The NP transport system for the comprehensive and synergistic roles of cancer nano-chemotherapy may be improved by means of attempts to co-govern drugs with ultrasound and photodynamic therapy and thermosensitive therapy [165,166].

#### 3.6.5. Personalized Therapy for Cancer

ανβ3-targeted para-magnetic NPs have been used to study angiogenesis in great detail, as well as to study nascent melanoma cancers non-intrusively, [167]. The efficacy of treatment, specifically in case of melanomas, can potentially arise through early detection [168]. To target malignant tumors with greater specificity and affinity, NPs associated with bio-targeting ligands, for example, small molecules, peptides and monoclonal antibodies, can be employed. In regard to each person’s molecular profile, these advancements offer opportunities for the development of tailored oncology, in which tumor therapy, diagnosis and detection are personalized [169].

Targeted towards tumors, tissue, cells, or organs, and governed by an external magnetic field, iron oxide NPs can be used to attach themselves with nucleotides, proteins, antibodies, and drugs. Towards cellular receptors such as urokinase plasminogen activator receptor, magnetic iron oxide NPs are currently being used. With the aim of improving the effectiveness of drug transport conducted by receptor-mediated endocytosis and for the destruction of cancer stromal fibroblasts, this nano-construct allows the immersion of drug-delivering NPs in the endothelial cell layer of the tumor, tissues, or cancerous cells. Due to their spatial imaging ability and increased biocompatibility, these iron oxide NPs may find significant applications in cancer treatment and imaging [170]. Breast cancer cells expressing HER2 receptors can be attached by means of the HER2 antibody (herceptin) trastuzumab, conjugated with iron oxide NPs. When attached to the tumor magnetically, it can exhibit increased antitumor activity with cytotoxic molecules such as doxorubicin [171].

#### 3.6.6. Cancer Treatment through Thermal Ablation

Metallic NPs have shown a capacity for implementation in targeted hyperthermic therapy, particularly in the case of carbon nanotubes, gold silica nanoshells, iron oxide nanoparticles, and solid gold NPs [172,173]. To treat deep tissue cancers, iron oxide NPs have been employed as both therapeutic and diagnostic nanoscale agents [174]. An increased amount of gold NPs could be obtained by marking gold NPs with antibodies in opposition to specific tumor cells. The application of radio-frequency fields to cells caused the confined heat and death of tumor cells, after the incorporation of NPs [172]. Through the utilization of magnetically induced heat, magnetic NPs provide an encouraging method for the minimally intrusive excision of small cancers in the breast [137,175]. This approach has the value of allowing the refined and selective tuning of the degree of energy deposition, permitting sufficient temperature control at the target site, and this method also meets the enhanced need for breast-conserving therapies [176]. The anti-human epidermal growth factor receptor 2 antibody can be employed in transporting drugs to human epidermal growth factor receptor 2-overexpressing tumors, and can initiate an anticancer response [175]. A pegylated colloidal GNP (gold nanoparticle) consisting of tumor necrosis factor-α attached to its surface called CYT-6091 has been shown to increase thermal therapies and has been broadly investigated as an adjuvant [177].

Furthermore, to treat tumors via the induction of hyperthermia, superparamagnetic NPs show attractive properties [178]. There is a transformation of magnetic energy into thermal energy when these NPs are subjected to an alternating magnetic field of adequate frequency and strength. The heat produced is then transported to the cells revolving around the NPs. Once the protein denatures and the restricted temperature increases more than 40 °C, this can cause tumor cell death via apoptosis [179,180].

### 3.7. Nanotechnology in the Treatment of Genetic Disorders

#### 3.7.1. Alzheimer’s Disease

Alzheimer’s disease (AD), the most prevalent form of dementia, is a neurodegenerative disorder [181,182], the initial symptoms of which include impaired memory and declining cognitive abilities, which lead to damage to the motor system [183]. A lot of literature supports the positive relationship between the concentration of the soluble aggregates of Aβ peptide and the degree of dementia in AD patients [184,185,186,187]. They accumulate to form insoluble fibrils, which further aggregate to form characteristic plaques [188,189]. Hence, most of the current research is centered towards the prevention of their aggregation. For this purpose, nanomaterials are exploited, owing to their exceptionally small size and fit for crossing the BBB.

Grape resveratrol (a neuroprotective, anti-inflammatory compound [190]) and OX26 mAB-conjugated solid lipid nanoparticles (SLNs) can inhibit Aβ aggregation [189]. SLNs have a hydrophobic lipid core, which allows the dispersion of the drug, thereby increasing its bioavailability [191,192]. Moreover, they are rapidly opsonized and clarified from the blood stream, which presents a convincing argument that these SLN do not accumulate in the blood stream unnecessarily, hence cutting down on the associated threats [193]. Similarly, the monoclonal antibody against fibrillary human amyloid β42 was conjugated with iron oxide NPs that successfully targeted aggregates in the arterioles of mice [194].

Curcumin and water-soluble PLGA-NPs conjugated with Tet-1 peptide can also destroy amyloid conjugates [195]. Curcumin, which has anti-mutagenic, anti-inflammatory, antioxidant, anti-cholesterol, anti-tau hyperphosphorylation, and anti-amyloid properties, is an excellent candidate for AD treatment [196,197,198,199,200,201,202]. B6 peptide was conjugated with curcumin-loaded PLGA-NPs [203], which decreased the diameter of curcumin, enhancing its cellular uptake. Additionally, they prevented tau hyperphosphorylation and deposition and boosted learning and memory in mice. Memantine, a neuronal death-preventing drug [204], has also been loaded into PEG-coated PLGA-NPs, which reduced β amyloid plaques and the characteristic inflammation of AD [205]. Negatively-charged gold nanoparticles (AuNPs) have also been proven to be effective against amyloids, since in their bare form they inhibited Aβ fibrillization and dissociated fibrils [206]. This finding marks them as potential carriers for anti-AD drugs. Protein capped cadium sulphate (PC-CdS) and iron oxide NPs exhibit anti-tau aggregation properties, while keeping the viability of neuroblastoma cells intact. Moreover, PC-CdS NPs can also disaggregate Tau cells [194,207].

#### 3.7.2. Parkinson’s Disease

Parkinson’s disease (PD) is the second most common neurodegenerative disease [208]. Dopaminergic (DA) neurons in the substantia nigra pars compacta selectively die and α-syn Lewy bodies start forming [209,210]. Six mutations are found to be behind familial PD—SNCA, DJ-1, parkin, PINK 1, ATP13A2, and LRKK2 [211]. Since α-syn’s connection to PD emergence has been established, scientists are now searching for new methods to interfere with its expression [212]. In a recent study, an *N*-isopropylacrylamide derivative was immobilized on oleic acid along with short hairpin RNA (shRNA) and loaded in magnetic iron oxide NPs. Nerve growth factor was also added to *N*-isopropylacrylamide. ShRNA interfered with α-syn synthesis successfully, thereby making it a potential tool for treating PD.

Retinoic acid (a neuroprotective chemical) NPs have also been used and found to be therapeutic for DA neurons. They also induced the production of mRNA and transcription factor proteins that make the survival of DA neurons possible, namely, Nurr 1 and PitX. This makes them suitable for use in the prevention of PD onset [213]. The co-loading of curcumin and piperine, which have extraordinary cognitive and antioxidant properties, on glycerly monoleate NPs has been performed recently. These NPs were coated with several surfactants that increased the bioavailability of loaded compounds. According to in vivo results, they can inhibit α-syn and reduce oxidative stress, apoptosis, toxicity induced by rotenone, and restrain DA’s neuronal degeneration process [214].

RNA interference (RNAi) can knockdown specific genes. In a study, α-syn-targeting RNAi and polyethylenimine NPs were used to treat incurable neurodegenerative disorders such as PD [215]. In a matter of 5 days, the α-syn level was reduced by almost 50% and no side effects (such as the induction of toxicity) were observed. PEG has also been exploited for use in the treatment of PD. PLGA-loaded NPs were coated with lactoferrin, which enhanced the delivery of loaded rotigotine. The results indicated that the cells that came in contact with it did not compromise their viability. In fact, free rotigotine was toxic. It was also reported that a high amount of rotigotine was heterogeneously distributed to the striatum, which is a primary affected region in PD [216].

#### 3.7.3. Amyotrophic Lateral Sclerosis

Amyotrophic lateral sclerosis (ALS) is another neurodegenerative disorder which is characterized by affected upper and lower motor neurons, the spinal cord, and the motor cortex region. Abnormal amounts of mutant superoxide dismutase (SOD) are also observed in ALS patients. The orderly progression of the disease has been explained by misfolded SOD 1. Hence, research is largely channeled towards reducing the levels of SOD [217]. Antisense oligonucleotides (ASOs) can effectively silence the proteins but their inability to cross the BBB rendered them useless. However, to overcome this problem, ASOs were loaded onto calcium phosphate lipid-coated NPs. When these were negatively charged, they successfully delivered ASO into a neuron-like cell line [218].

Oxidative stress and damage have also been reported to contribute to ALS. Reactive oxygen species (ROS) and reactive nitrogen species (RNS) damage DNA, RNA, and other molecules [219]. Cerium NPs can take part in coupled redox reactions that neutralize ROS and RNS. This makes them suitable for use in antioxidant therapy for a number of neurodegenerative disorders, including ALS [220].

#### 3.7.4. Huntington’s Disease 

Huntington’s disease (HD), an autosomal-dominant neurodegenerative disorder, is characterized by anxiety, involuntary movements, and chorea [221,222]. It has been observed that biological materials obtained from patients with neurodegenerative diseases such as Huntington’s exhibit oxidative stress and mitochondrial defects. HD brains have faulty electron transport chains as well [223]. 3-nitroproponoic acid (3-NP) is a neurotoxin which leads to the generation of ROS; therefore, researchers are looking for new agents that can potentially inhibit the production of 3-NP.

Curcumin has also been used to treat Huntington’s disease, along with SLN. They can ameliorate 3-NP induced in HD mice by decreasing the amount of intermediate complex II activity. The signs observed include reduced swelling of the mitochondria, lipid peroxidation, and ROS production. Neuromotor coordination was also enhanced [224]. SLNs have been loaded with rosmarinic acid and introduced into 3-NO-induced mice and this also showed promising results [225]. β cyclodextrin (CD) NPs have also been used to carry siRNA, which can silence or modify the expression of mutant HTT. CDNPs decreased the amount of mutant gene mRNA dramatically and also showed partial toxicity, but the overall toxicity profile was satisfying [226].

Trehalose-loaded zwitterion NPs inhibit amyloid and polyglutamine aggregation in HD mice brains [227]. Their zwitterionic shell enhances cell uptake without inducing cytotoxicity. It hindered aggregation by forming multivalent bonds. This method requires trehalose in micro amounts; however, when used in molecular form it is needed in milli-molar concentrations. Trehalose and zwitterion are other anti-amyloidogenic molecules.

#### 3.7.5. Cystic Fibrosis

Cystic fibrosis is a fatal genetic disorder caused by a mutation in the cystic fibrosis transmembrane conductance (CFTR) gene. It is characterized by abnormal transportation in endothelium cells of a number of tissues. This leads to abnormally thick and sticky mucus, which blocks organs. The chief blocked organ is the lung. This translates into the emergence of recurrent bacterial infections, which destroy the lung tissues progressively, and as a result, pulmonary disease grows large enough to cause mortality [228,229]. To treat this disease, correction of the CFTR gene seems like an attractive option.

Chemically altered mRNA was loaded onto lipid NPs. It was reported that this allowed an increase in the number of membrane localized CFTR, restoring its primary function of acting as a chloride channel. Additionally, the nasal application of these NPs restarted chloride transport to nasal airway epithelium cells. This proved to be an effective tool for CFTR treatment that can be manipulated in the future.

PLGA-NPs coated with PEG have been employed for almost every genetic/neurodegenerative disorder, including cystic fibrosis as well. In this case, they effectively delivered anti-inflammatory compounds and CFTR correctors [230]. Another interesting approach employed for the treatment of CFTR infections was loading biodegradable NPs with antibiotics, such as ciprofloxacin complex. This approach was adopted to fight the infections that are characteristic of the disease itself. The bacteria which were targeted by these antibiotic-loaded NPs were *Pseudomonas aeruginosa*. Mucus was checked and analyzed for results after treatment. It was reported that colloidal stability was proven and that the mucus became noticeably less turbid, showing a decrease in pathogenic bacteria [231]. Table 2 summarizes the outcomes of some nanomaterials employed to treat various genetic disorders. 

### 3.8. Nanotechnology in the Treatment of Nervous System Diseases 

Fortunately, NPs are not only valuable for genetic or neurodegenerative disorders; they have also been manipulated to treat other severe neurological traumas, such as in post-stroke neuroprotection and spinal cord injuries. These two areas might sound very complex, but studies have proven over time that nanotechnology can help to fight these severe diseases. Regeneration or repair in the central nervous system is another large problem. This is a prevalent problem because the damaged axons lack the ability to regenerate and regrow. The obstacles in the way of the regeneration of these axons are extrinsic inhibitory molecules and an age-dependent drop in intrinsic regenerative capacity, along with some other factors [232,233]. Presently, researchers are investing their efforts in looking for alternative ways to inhibit the action of factors that do not promote the growth and regeneration of damaged axons and neural cells. In this regard, nanotechnology is found to be a highly effective potential tool to treat central nervous system disorders [234].

For the treatment of spinal cord injuries, conventional drugs are used, which have become unpopular due to drawbacks associated with them. These drugs, when systematically administered, were found to be highly inefficient since they were metabolized rapidly before reaching the target and were cleared from the bloodstream. Now, the aim is to modify them in such a way that their bioavailability can be enhanced. For this purpose, adenosine was conjugated with lipid squalene into nano-assemblies. This method showed astonishing results, since the neurologic deficit score was improved and early motor recovery of the hind limbs was also observed [235].

Macrophages have been observed to perform a key role in the entire inflammation process in microglia and macrophages and they contribute to the chronic phase of neurodegeneration; hence, they have been established as a therapeutic target [236]. Polymethyl methacrylate NPs have been used to target specific cell populations of macrophages in order to decrease inflammation without exhibiting toxicity [237]. A charged surface and surface PEGylation enhance this process, allowing cellular uptake. This is a different approach for the treatment of such diseases. Table 3 summarizes the outcomes of some nanomaterials employed with and without drugs in an effort to treat some major neurodegenerative disorders.

## 4. Conclusions and Future Perspectives

Nanotechnology research has grown exponentially within the last few decades, and the focus on healthcare sectors has increased in parallel. Theranostic development has led to a significant amount of understanding of some of the complex etiologies involved, as well as increasing the chances of early diagnosis and therapeutic potential with the help of nanomedicine. Various nanosystems have been exploited and integrated at a limited scale but have proven to be efficient in solving various bottlenecks in various healthcare sectors. However, nanomedicines and nanodevices are still at an early developmental stage and one way to accelerate this process is to direct research studies so that researchers work towards developing new methods to overcome the associated limitations. The gradual development of nanotechnology-based methods has given rise to a hope that soon life-threatening and disabling disorders will be effectively treated. The gaps due to inadequate efficacy and preclinical safety studies need to be filled on a priority basis so that we can make full and timely use of the great potential of nenotechnology, which is yet to be realized. Nanotechnology has a solution to many problems, but this does not mean in any way that there are no challenges or limitations associated with it.

One of the major obstacles in the implementation of nano-based products in living systems for healthcare services is toxicity. Various nanomaterials have triggered unwanted allergic and other reactions that can be potentially harmful to the body. Toxicity is a very complex concept in itself because it is dependent on a diverse range of factors such as morphology, size, dose, surface area, route, and duration of administration [246]. This directs our attention towards another area, which is the need to standardize or personalize the use of nanomaterials. Furthermore, the reliability and reproducibility of experiments involving NPs remains another area that needs to be worked on. Since these are extremely small entities, controlling their activity in sensitive environments is also hard. Some other limitations are their high cost, the presence of impurities, their environmental impacts, etc. [247]. If these dangers are not dealt with carefully, they may have seriously lethal repercussions.

Since nanotechnology is a relatively new area, it remains relatively underexplored. In fact, we cannot say that the physiochemical behavior of these NPs is fully known in vivo and in vitro. This is why we might not judge accurately that which type of nanomaterial will be used precisely for what purpose. There are some NPs that might be very useful in one system, but in other systems they might be entirely toxic. One example of such a case is PEI, which is an excellent transporter of nucleic acids, but it shows cytotoxic traits [237]. Another factor that we tend to ignore is that the NPs have varying compositions, sizes, and shapes and each of them has different impacts on living systems. Moreover, the duration exposure, as well as the coating, aggregation, charge and solubility of nanomaterials, also influence their performance [237].

Despite the sophisticated instruments and tools developed in recent times, we still need to develop modern tools that can quickly characterize synthetic nanomaterials, separately from existing analytical tools. We are also in dire need of establishing standardized protocols to synthesize these nanomaterials, not only ensuring high yields, stability, and purity, but also complying with the issued security guidelines. An efficient in vivo monitoring system can considerably boost biomedical processes such as the treatment and diagnosis of various serious diseases [248]. There is also a need to come up with mechanisms that help us to thoroughly understand the fate of NPs once they have been used. These questions include how long they stay in the body, what conditions impact the duration of degradation, how to make them stay for longer and shorter periods, what are their long-term and short-term impacts, how exactly does the body behave towards these outsider entities on a micro and a macro level, what are their characteristics and their mechanisms of action, and how we can standardize these particles to ensure the reproducibility of experiments. These should be addressed before the implementation of nanotechnologies in healthcare sectors. Apart from these, there are also many questions that require well-studied and well-experimented answers. We also need to identify the potential hazards associated with these nanomaterials in order to avoid any unforeseen circumstances. Furthermore, the different nanomedicines and nanoformulations targeting various diseases must be meticulously designed in order to achieve the safest and most efficacious therapeutic regimen. We conclude with the vision that nanotechnology will push forward to develop more promising therapies to cope with various severe diseases, and will also provide researchers with effective tools to solve the various bottlenecks in healthcare sectors.

## Figures and Tables

**Table 2:** Summary of the various nanosystems used for treating genetic disorders.

Genetic Disease	Nanosystem	Effects	Ref.
**Alzheimer’s Disease**	Solid lipid nanoparticles (SLNs) PLGA NPs conjugated with Tet-1 peptide	inhibited Aβ aggregation improved cellular uptake	[189,190,205]
**Parkinson’s Disease**	Retinoic acid NPs	Increased bioavailability of loaded compounds, prevented apoptosis, oxidative stress, and toxicity	[213,214]
**Amyotrophic Lateral Sclerosis**	Calcium phosphate lipid-coated NPs	able to pass through the BBB, neutralized RNS and ROS reactions	[218,219]
**Huntington’s Disease**	Curcumin SLN	Reduced the activity of intermediate complex II	[224,227]
	Trehalose-loaded zwitterion NPs	Inhibited amyloid and polyglutamine aggregation	
**Cystic Fibrosis**	Lipid NPs	Increased the amount of membrane-localized CFTR	[230,231]
	PLGA NPs coated with PEG	Effectively delivered anti-inflammatory compounds	

**Table 3:** Summary of the various nanosystems used for the treatment of central nervous system disorders.

Disease	Nanosystems Used	Drug	Target	Effect	Reference
**Optic Nerve Injury**	Designed, self-assembling peptide nanofiber	None	Site of acute injury in axon and dismantled brain tissues of hamsters	Creates permissive environment for axon regeneration and knitting brain tissues	[238]
**Optic Nerve Injury**	Self-assembling peptide nanofiber scaffold	None	Chronic optic tract (OT) lesion and damaged axons of hamsters	Both show improved healing activity	[239]
**Optic Nerve Injury**	Fabricated PLGA-coated NPs	EGFR TKI 4-(3-chloroanilino)-6,7-dimethoxyquinazoline (AG1478)	Rat optic nerve crush injury	Optic nerve regeneration	[240]
**Optic Nerve Injury (Glaucoma)**	PLGA nanosphere	Ciliary neurotrophic factor (CNTF)	Retinal ganglion cells (RGCs)	Prolonged survival of RGCs in rats	[241]
**Optic Nerve Injury**	PLA nanoparticles	water soluble mitomycin C (MMC)	Tumor tissues	Inhibited scar formation	[242]
**Spinal Cord Injury**	Peptide amphiphile (PA) self-assembling nanofibers	None	Astrogliosis process	Reduced astrogliosis and cell death, increased number of oligodendroglia at the site of injury, promoted regeneration of descending motor fibers and ascending sensory fibers	[243]
**Spinal Cord Injury**	carboxymethylchitosan/polyamidoamine (CMCht/PAMAM) dendrimer	Methylprednisolone	Glial cells	significant differences in the locomotor output	[244]
**Stroke and Spinal Cord Injury**	Squalenoyl adenosine NPs	None	Fast metobolizing rate of neuroprotective adenosine	prolonged circulation of this nucleoside, to provide neuroprotection in mouse stroke and rat spinal cord injury	[236]
**Spinal Cord Injury**	Polymeric NPs	None	Circulating immune cells	immune cell infiltration is reduced, leading to decreased tissue degeneration	[245]

## Data Availability

Data sharing not applicable.
